# Nature of Bimetallic Oxide Sb_2_MoO_6_/rGO Anode for High‐Performance Potassium‐Ion Batteries

**DOI:** 10.1002/advs.201900904

**Published:** 2019-06-18

**Authors:** Jue Wang, Bin Wang, Zhaomeng Liu, Ling Fan, Qingfeng Zhang, Hongbo Ding, Longlu Wang, Hongguan Yang, Xinzhi Yu, Bingan Lu

**Affiliations:** ^1^ School of Physics and Electronics State Key Laboratory of Advanced Design and Manufacturing for Vehicle Body Hunan University Changsha 410082 P. R. China; ^2^ Physics and Electronic Engineering Department Xinxiang University Xinxiang 453003 P. R. China; ^3^ Fujian Strait Research Institute of Industrial Graphene Technologies Quanzhou 362000 P. R. China

**Keywords:** anodes, bimetallic oxide, density functional theory (DFT) calculation, operando X‐ray diffraction, potassium‐ion batteries

## Abstract

Potassium‐ion batteries (KIBs) are one of the most appealing alternatives to lithium‐ion batteries, particularly attractive in large‐scale energy storage devices considering the more sufficient and lower cost supply of potassium resources in comparison with lithium. To achieve more competitive KIBs, it is necessary to search for anode materials with a high performance. Herein, the bimetallic oxide Sb_2_MoO_6_, with the presence of reduced graphene oxide, is reported as a high‐performance anode material for KIBs in this study, achieving discharge capacities as high as 402 mAh g^−1^ at 100 mA g^−1^ and 381 mAh g^−1^ at 200 mA g^−1^, and reserving a capacity of 247 mAh g^−1^ after 100 cycles at a current density of 500 mA g^−1^. Meanwhile, the potassiation/depotassiation mechanism of this material is probed in‐depth through the electrochemical characterization, operando X‐ray diffraction, transmission electron microscope, and density functional theory calculation, successfully unraveling the nature of the high‐performance anode and the functions of Sb and Mo in Sb_2_MoO_6_. More importantly, the phase development and bond breaking sequence of Sb_2_MoO_6_ are successfully identified, which is meaningful for the fundamental study of metal‐oxide based electrode materials for KIBs.

## 1 Introduction

It is debatable that whether the lithium reserves on the earth, ≈14 million tons, are able to satisfy the rapidly expanding markets of electric vehicles and grid‐level energy storage systems, in addition to an uneven distribution of lithium resources as well as the relatively high cost of lithium‐ion batteries (LIBs).^1^, ^2^, ^3^, ^4^, ^5^ Because of the natural abundance of potassium, a similar redox potential of potassium to lithium, and the low cost, potassiumion batteries (KIBs) serve as a promising substitution to LIBs,^6^, ^7^, ^8^, ^9^, ^10^, ^11^ especially attractive in the large‐scale energy storage systems which strive intensively to lower the price to be competitive with other energy storage techniques. However, in comparison with the smaller lithium ions (a radius of 0.76 Å), the large‐sized potassium ions (a radius of 1.38 Å) could arouse a severe volume change of the electrode material during charge/discharge, seriously weakening the electrode stability and resulting in an unsatisfied battery performance.^12^, ^13^, ^14^, ^15^, ^16^ Therefore, searching for the high performance KIBs anode (a critical component of KIBs) to alleviate the dramatic volume change is highly demanded to build high performance KIBs.

Besides the carbonaceous anodes (graphite, soft carbon, hard carbon, etc.) which are being explored vigorously but yield a relatively low specific capacity,^17^, ^18^, ^19^, ^20^, ^21^, ^22^, ^23^, ^24^, ^25^, ^26^, ^27^ metal oxides, such as iron oxides,^28^ molybdenum oxides,^29^, ^30^ niobium pentoxides,^31^ tin oxides,^32^ and titanium oxides,^33^ are interesting anode candidates considering their high gravimetric and volumetric specific capacity, which are able to provide high performance anodes for KIBs.^34^, ^35^ For example, antimony oxide (Sb_2_O_3_) possesses a theoretical capacity as high as 1103 mAh g^−1^ for KIBs through both conversion reactions and alloy reactions. Nevertheless, it suffers from the poor electronic conductivity as well the enormous volume variation during charge/discharge, giving rise to the upset rate performance and cycling stability,^36^ which certainly needs to be resolved.

It is usually expected that bimetallic compounds could display a superior battery performance than the single‐metal counterparts because of the synergistic effects, interfacial effects, and more active sites.^37^, ^38^, ^39^, ^40^ Sb_2_MoO_6_, a typical bimetallic oxide, has been explored as anode materials for lithium‐ and sodium‐ion batteries very recently, which demonstrates a promising battery performance.^41^, ^42^, ^43^, ^44^ It is discovered that the alloying and dealloying reactions between Sb and Li/Na provide the majority of the reversible capacity, while the production of Li_2_O/Na_2_O–MoO*_x_* mitigates the volume expansion through offering a buffering matrix. Therefore, Sb_2_MoO_6_ is believed to be very encouraging anode candidate for KIBs, since the two Sb atoms within the chemical formula are able to offer a theoretical capacity as high as 738 mAh g^−1^, while the Mo may help enhance the stability of the electrode by forming a buffering matrix as well as improve the conductivity, which has not been reported in KIBs yet. Moreover, the insertion of potassium ions, which are considerably larger than lithium or sodium ions, may intrigue the quite different phenomena in Sb_2_MoO_6_ crystal from the intercalation of lithium or sodium ions, requiring a fundamental study to explore.

Herein, Sb_2_MoO_6_/reduced graphene oxide (rGO) composites were facilely prepared in this study using a hydrothermal method and explored as the anode materials for KIBs, which exhibit capacities as high as 402 mAh g^−1^ at a current density of 100 mA g^−1^ and 381 mAh g^−1^ at 200 mA g^−1^, and maintain a capacity of 247 mAh g^−1^ over 100 cycles at 500 mA g^−1^. To determine the functions of Sb and Mo in Sb_2_MoO_6_ and profoundly understand the potassium‐storage mechanism, Sb‐ and Mo‐based oxides were fabricated following the identical procedures as well. Moreover, electrochemical tests, operando X‐ray diffraction (XRD) measurements, transmission electron microscope (TEM) characterizations, and density functional theory (DFT) calculations were carefully performed to probe the nature of Sb_2_MoO_6_/rGO anode. It is demonstrated that Sb_2_MoO_6_/rGO composites show a largely enhanced rate performance and cycling stability in comparison with the Sb‐ and Mo‐based oxides/rGO. The nature of this high‐performance anode material and roles of these two metallic elements, Sb and Mo, are studied. More remarkably, the phase evolution and bond breaking sequence of Sb_2_MoO_6_ during charge/discharge are successfully deciphered, which sheds light on the potassium‐storage mechanism of this type of electrodes. This research could serve as a valuable fundamental study which benefits the advancement of KIBs in metal‐oxide based electrode materials.

## 2 Result and Discussion

**Figure 1:**
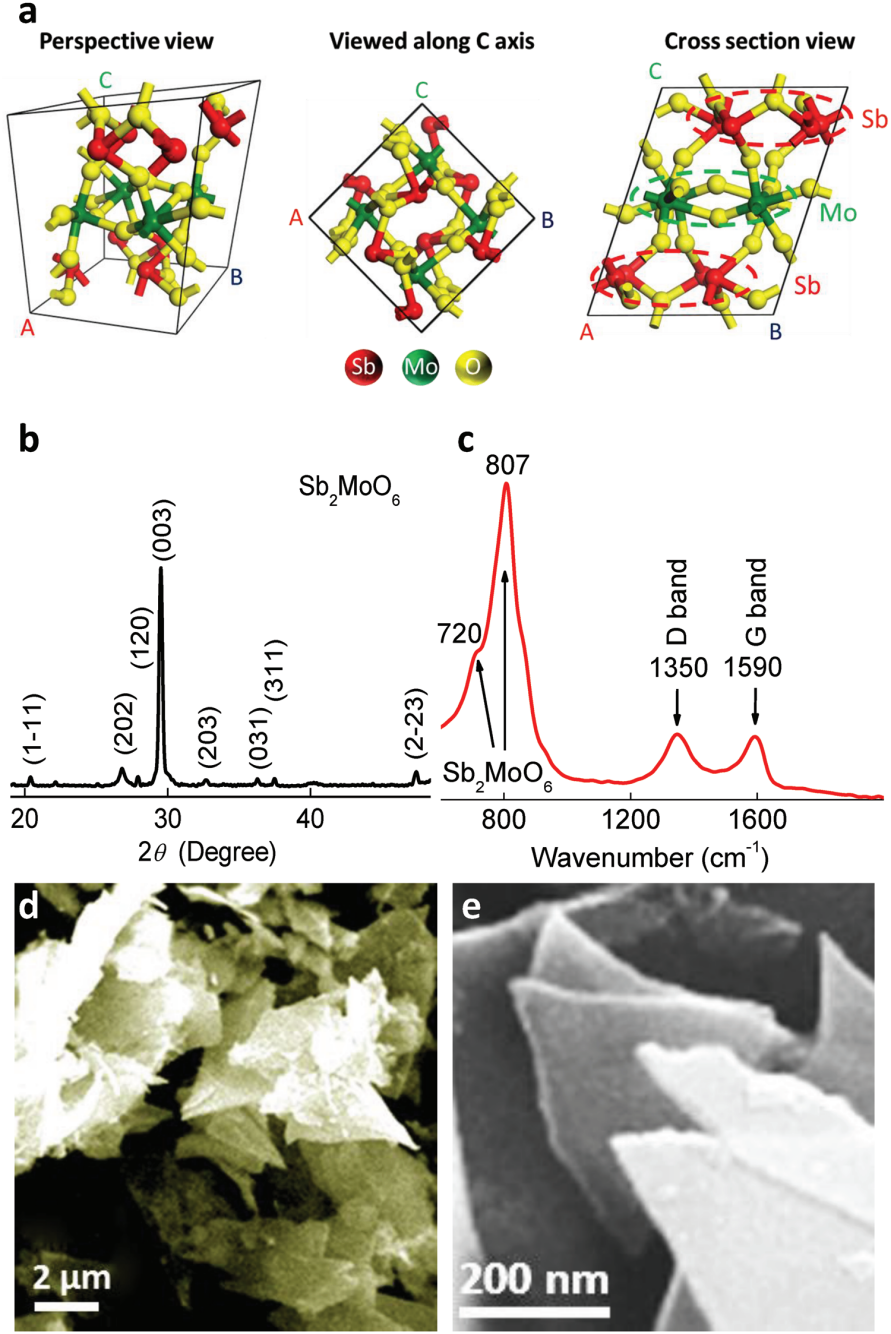
a) Crystal structure of Sb_2_MoO_6_. b) XRD pattern, c) Raman spectra, d) low resolution, and e) high resolution SEM images of Sb_2_MoO_6_/rGO composites.

The lattice structure of typical Sb_2_MoO_6_ (P1 space group) is presented in **Figure** **1**a in the perspective view, top view along *C* axis, and cross‐section view. There are four Mo atoms located at the center of the crystal unit, with each four Sb atoms residing above and below the layer of Mo atoms. To figure out the roles of Sb and Mo within Sb_2_MoO_6_ during the potassiation/depotassiation, Sb_2_MoO_6_ nanostructures were synthesized using a simple hydrothermal method in this study, with SbCl_3_ as the Sb sources, Na_2_MoO_6_ as the Mo source, and graphene oxide (GO) nanosheets as the template to induce the growth of nanoplates. The crystal structure of as‐prepared Sb_2_MoO_6_ samples was characterized via XRD, as demonstrated in Figure 1b. The XRD pattern matches well with Sb_2_MoO_6_ (PDF#75‐1236), which belongs to a triclinic structure of P1 space group, and the corresponding facets are labeled in Figure 1b. There is a distinctive peak around 29.5°, which is indexed to the (003) facet and it is much more intense than other peaks, indicating the good crystallinity of Sb_2_MoO_6_. From the Raman spectra in Figure 1c, these products are also confirmed to be Sb_2_MoO_6_, displaying the characteristic peaks around 720 and 810 cm^−1^. Meanwhile, the appearances of D band around 1350 cm^−1^ and G band near 1590 cm^−1^ illustrate the existence of rGO, a reduction product of GO which was added during synthesis to induce the generation of Sb_2_MoO_6_ nanoplates and avoid the aggregation of these nanoplates.^42^ Meanwhile, the presence of rGO could enhance the electrical conductivity and thus improve the rate performance of Sb_2_MoO_6_ for potassium storage.^42^, ^45^ Given that rGO should exist in the Mo‐ and Sb‐based counterparts, since these counterparts are prepared using the same experimental procedures as Sb_2_MoO_6_/rGO with the addition of GO, rGO will not cause interference to the comparison of the potassium storage performance between them. It is worth mentioning that no obvious diffraction peaks from rGO can be detected in the XRD pattern, possibly attributed to a small amount and/or the poor crystallinity of rGO. The morphology of as‐prepared nanostructure is revealed by scanning electron microscope (SEM) images in Figure 1d,e, displaying a well‐defined plate shape. Based on the atomic force microscopy (AFM) image and the corresponding height profile (Figure S1, Supporting Information), with two nanoplates stacked, a total height of 154 nm is obtained, indicating a thickness around 77 nm for these Sb_2_MoO_6_ nanoplates. Sb_2_MoO_6_ was also prepared using the same procedures without the addition of GO during the synthesis. With the absence of GO, nanobelts were developed, as shown in Figure S2 (Supporting Information), validating that GO was working as a template to induce the growth of Sb_2_MoO_6_ nanoplates, in agreement with the literature.^42^

**Figure 2:**
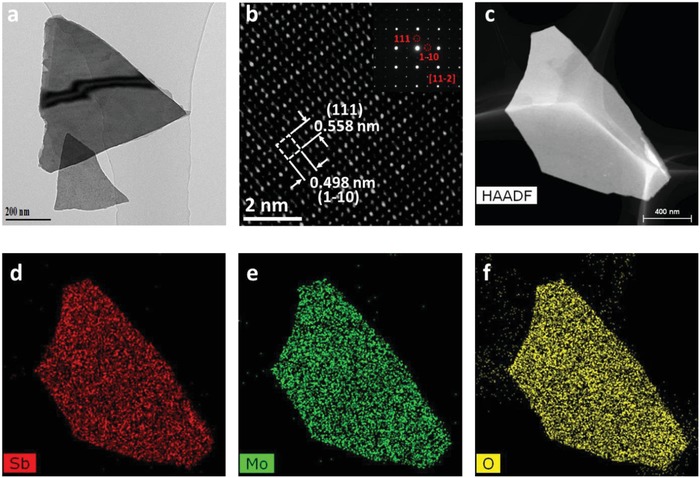
a) TEM image, b) HRTEM image, c) HAADF‐STEM image, and d–f) EDS mappings of Sb_2_MoO_6_ nanoplates.

The morphology of the Sb_2_MoO_6_ nanoplate is further verified by TEM image in **Figure** **2**a, illustrating clear nanoplates. In addition, rGO nanosheets are also detected in the TEM images (Figure S3, Supporting Information), verifying a product of Sb_2_MoO_6_/rGO composites. The high resolution transmission electron microscope (HRTEM) image (Figure 2b) and selected area electron diffraction image shown in the inset present the lattice information of Sb_2_MoO_6_ nanoplates, displaying lattice distances of 0.555 and 0.396 nm which correspond to (111) and (1‐10) facets, respectively. To obtain the elemental distribution of the nanoplate, high‐angle annular dark‐field imaging (HAADF)‐scanning transmission electron microscope (STEM) as well as energy dispersive X‐ray spectra (EDS) elemental mappings were carried out and the results are shown in Figure 2c–f. The compositions of the Sb_2_MoO_6_ nanoplates are elucidated to be Sb, Mo, and O distributed uniformly through the whole nanoplate. To interpret the functions of Sb and Mo in the high‐performance Sb_2_MoO_6_/rGO anode for KIBs, Mo‐ and Sb‐based counterparts were synthesized following the identical experimental protocols, but excluding Mo source (Na_2_MoO_4_) for the Sb‐based counterpart while removing the Sb source (SbCl_3_) for the Mo‐based counterpart. According to the XRD patterns (Figure S4, Supporting Information), TEM images, HAADF‐STEM images, and EDS mappings (Figure S5, Supporting Information), Sb_2_O_3_/Sb_8_O_11_Cl_2_ nanoplates are identified for Sb‐based counterpart and MoO_2_ nanoplates are recognized for Mo‐based counterpart. It is worth mentioning that rGO should also exist in the Mo‐ and Sb‐based counterparts, since the same experimental procedures were adopted in preparing these counterparts with the addition of GO, which is not expected to interfere the comparison of the electrochemical performances between Sb_2_MoO_6_/rGO and the counterparts.

**Figure 3:**
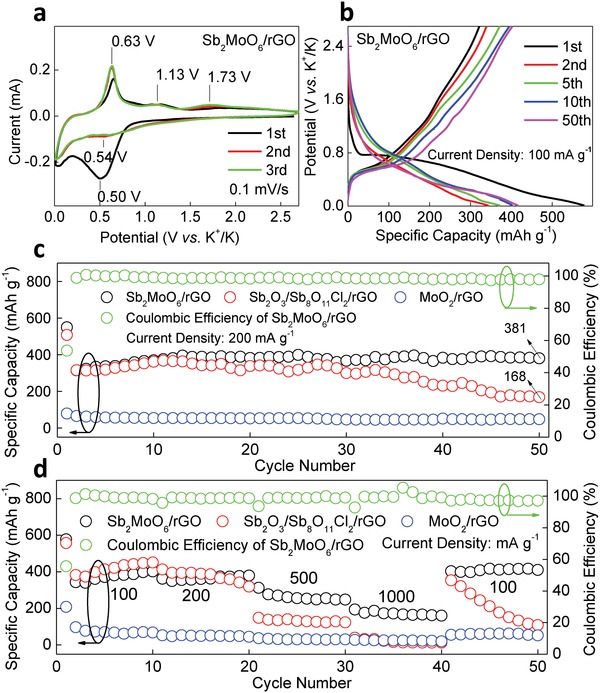
a) CV of Sb_2_MoO_6_/rGO. b) Charge/discharge curves of Sb_2_MoO_6_/rGO at 100 mA g^−1^. c) Cycling performance of Sb_2_MoO_6_/rGO, Sb_2_O_3_/Sb_8_O_11_Cl_2_/rGO, and MoO_2_/rGO at 200 mA g^−1^. d) Rate performance of Sb_2_MoO_6_/rGO, Sb_2_O_3_/Sb_8_O_11_Cl_2_/rGO, and MoO_2_/rGO.

To investigate the electrochemical properties of Sb_2_MoO_6_/rGO, cyclic voltammetry (CV) profiles were executed in the half cell with a scanning rate of 0.1 mV s^−1^ and the curves of initial three scans are depicted in **Figure** **3**a. In the measurements, the studied material serves as the working electrode and the potassium metal functions as the counter and reference electrodes, with an electrolyte of 3 m potassium bis(fluorosulfonyl)imide (KFSI) in dimethyl ether (DME). The option of the KFSI electrolyte is according to the previous study,^46^, ^47^, ^48^ which could form a highly stable inorganic solid‐electrolyte interphase (SEI) layer that is different from the SEI film primarily composed of organic components generated from the traditional KPF_6_ electrolytes, suppressing the development of potassium dendrites, avoiding excessive side reactions, and reducing the polarization. Therefore, this kind of electrolyte may provide a better performance for potassium‐ion based energy storage devices. In the first cathodic scan, a broad peak appears at 0.50 V, which is primarily associated with the intercalation of K^+^ into Sb_2_MoO_6_ crystal, formation of SEI, and reduction of Sb^3+^ to Sb. In the following scans, a shallow peak stays close to 0.54 V, corresponding to the alloying reactions between Sb and K^+^. In the anodic scans, there are three peaks located at 0.63, 1.13, and 1.73 V, designated to the dealloy reactions. The anodic peak at 0.63 V becomes stronger after the first cycle, which could be due to an activation process at the beginning. CV profiles of the Sb‐ and Mo‐based counterparts are shown in Figure S6 (Supporting Information). The Sb‐based counterpart (Sb_2_O_3_/Sb_8_O_11_Cl_2_/rGO) exhibits a strong cathodic peak around 0.25 V in the first scan, which is due to the SEI formation and conversion reactions. In the following scans, an intense cathodic peak located at 0.54 V around is witnessed, the same location as Sb_2_MoO_6_/rGO. The location and shape of anodic peaks of Sb_2_O_3_/Sb_8_O_11_Cl_2_/rGO at 0.67, 1.17, and 1.63 V are highly analogous to those of Sb_2_MoO_6_/rGO. For the Mo‐based counterpart MoO_2_/rGO, there are two broad peaks in the first scan, a cathodic peak around 0.68 V and an anodic one close to 1.23 V, which are attributed to the decomposition of the electrolyte in the formation of SEI film. In the subsequent scans, these two peaks disappear. However, a strong cathodic peak of the MoO_2_/rGO composites around 0.01 V corresponding to the insertion of K^+^ into the K–Mo–O matrix and an anodic peak located at about 0.29 V indexed to the extraction of K^+^ from the K–Mo–O matrix are retained.^29^, ^30^

The typical charge/discharge curves of Sb_2_MoO_6_/rGO electrode at a current density of 100 mA g^−1^ are shown in Figure 3b. In the first cycle, it delivers a discharge capacity of 578 mAh g^−1^ and a reversible charge capacity of 322 mAh g^−1^, equivalent to an initial Columbic efficiency of 55.7 %. The second discharge capacity drops to 344 mAh g^−1^. The large difference of the discharge capacity between the initial cycle and the following cycle is caused by the formation of SEI film. With the test of battery proceeding, the discharge capacity increases, reaching a capacity above 400 mAh g^−1^ in the 10th cycle. The increase of capacity suggests the existence of an activation process for Sb_2_MoO_6_/rGO. In the 10th cycle, a clear discharge plateau around 0.45 V can be observed, which is consistent with the locations of cathodic peaks in CV scans, correlating to the alloying reactions between Sb and K^+^. The plateau around 0.45 V is skewed by the decomposition of electrolyte in the formation of SEI film, destruction of Sb_2_MoO_6_ lattice, and reduction of Sb^3+^ to Sb in the first few cycles, which makes it difficult to observe. With those processes approaching the end, the plateau around 0.45 V turns clear in the 10th cycle. With the cycling proceeding, the platform is retained, which is obviously shown in the 50th cycle. The variation of the plateau before and after the 10th cycle means that the decomposition of electrolyte in the formation of SEI film, destruction of Sb_2_MoO_6_ lattice, and reduction of Sb^3+^ to Sb primarily occur in the first few cycles. There is an obvious charging plateau around 0.6 V in the charge/discharge curves, agreeing with the intense anodic CV peak around 0.63 V which is related to the dealloying reactions.

The charge/discharge curves of Sb_2_O_3_/Sb_8_O_11_Cl_2_/rGO and MoO_2_/rGO composites at 100 mA g^−1^ are described in Figure S7a,b (Supporting Information), respectively. In addition to the profiles akin to that of Sb_2_MoO_6_/rGO, Sb_2_O_3_/Sb_8_O_11_Cl_2_/rGO composites display a similar activation process with even higher capacities than that of Sb_2_MoO_6_ nanoplates. On the contrary, MoO_2_/rGO composites only provide a low reversible discharge capacity, which quickly decreases to 66 mA g^−1^ in the 5th cycle. The low specific capacity of MoO_2_/rGO, which is not expected to be from the conversion reaction of the Mo element or the alloy reaction between the Mo element and K^+^, should be due to the insertion/extraction of K^+^ in the K–Mo–O matrix, consistent with the CV results mentioned above. Therefore, it is deduced that the Mo element may not involve in the conversion reaction or alloy reaction with K^+^, but provides a low specific capacity through the insertion/extraction of K^+^ in the K–Mo–O matrix. The cycling performances of Sb_2_MoO_6_/rGO, Sb_2_O_3_/Sb_8_O_11_Cl_2_/rGO, and MoO_2_/rGO composites at 200 mA g^−1^ are revealed in Figure 3c. It shows an activation process for Sb_2_MoO_6_/rGO as the discharge capacity gradually increases at the beginning. A high discharge capacity of 381 mAh g^−1^ is retained after 50 cycles. On the contrary, even though the discharge capacity of Sb_2_O_3_/Sb_8_O_11_Cl_2_/rGO grows initially, it falls quickly after 30 cycles, leaving only 168 mAh g^−1^ after 50 cycles. MoO_2_/rGO composites present a flat cycling performance with a low capacity around 48 mAh g^−1^ over 50 cycles. Under a relatively large current density of 500 mA g^−1^, the cycling performances of Sb_2_MoO_6_/rGO, Sb_2_O_3_/Sb_8_O_11_Cl_2_/rGO, and MoO_2_/rGO are displayed in Figure S8 (Supporting Information). Although Sb_2_O_3_/Sb_8_O_11_Cl_2_/rGO composites reach a high capacity around 280 mAh g^−1^ at the beginning, only 51 mAh g^−1^ is left after 100 cycles, in comparison to a capacity of 247 mAh g^−1^ retained for Sb_2_MoO_6_/rGO, indicating a much superior cycling performance of Sb_2_MoO_6_/rGO. MoO_2_/rGO composites only offer a low capacity around 40 mAh g^−1^ over 100 cycles.

The comparison of the rate performances of Sb_2_MoO_6_/rGO, Sb_2_O_3_/Sb_8_O_11_Cl_2_/rGO, and MoO_2_/rGO is presented in Figure 3d. Sb_2_MoO_6_/rGO composites achieve a high discharge capacity of 402 mAh g^−1^ at 100 mA g^−1^, and then deliver capacities of 350, 248, and 161 mAh g^−1^ with a high Coulombic efficiency when the current density grows to 200, 500, and 1000 mAh g^−1^. Once the current density returns to 100 mA g^−1^, it recovers to an impressive discharge capacity of 403 mAh g^−1^, expressing an excellent rate capability. Alike Sb_2_MoO_6_/rGO composites, Sb_2_O_3_/Sb_8_O_11_Cl_2_/rGO composites achieve a discharge capacity as high as 451 mAh g^−1^ at 100 mA g^−1^, but it rapidly drops to 319, 124, and 13 mAh g^−1^, as the current density increases to 200, 500, and 1000 mAh g^−1^, respectively. After the current density is reduced to 100 mA g^−1^, the discharge capacity is back to 354 mAh g^−1^, which is 21.5% lower than the 451 mAh g^−1^ prior to experiencing the large current density. Meanwhile, its discharge capacity quickly dives to 109 mAh g^−1^ after just 9 cycles. It is apparent that Sb_2_O_3_/Sb_8_O_11_Cl_2_/rGO composites possess a disappointing rate capability, which is due to a low conductivity and electrode instability. MoO_2_/rGO composites offer low discharge capacities of 69, 45, 29, 20 mAh g^−1^ at current densities of 100, 200, 500, and 1000 mAh g^−1^, respectively, and the current density only recovers to 56 mAh g^−1^ once the current density is back to 100 mA g^−1^, stating a low capacity at all current densities. From the above electrochemical results, it can be concluded that the high capacity of Sb_2_MoO_6_/rGO composites for KIBs is majorly from the Sb element while the existence of Mo element enhances the conductivity and stability.

To further probe the electrochemical process, electrochemical impedance spectra (EIS) of fresh electrode of Sb_2_MoO_6_/rGO composites, as well as the same electrode after being cycled at 500 mA g^−1^ for 10 and 50 cycles, were measured from 0.01–100 kHz with an amplitude of 5 mV. The outcomes are illustrated using the Nyquist plots (Figure S9, Supporting Information). After being cycled at 500 mA g^−1^ for 10 times, the diameter of the semicircle in the middle frequency, which characterizes the charge‐transfer resistance (*R*_ct_), declines considerably, expressing the formation of SEI film in the early period. However, *R*_ct_ only changes slightly after 50 cycles, elucidating the formation of a relatively stable SEI film, which benefits the stability of the battery performance.

**Figure 4:**
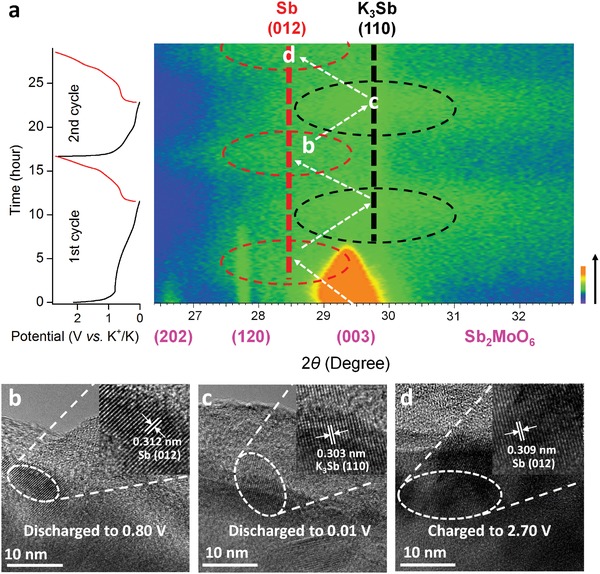
a) Contour plot of the operando XRD results of the Sb_2_MoO_6_/rGO during potassiation/depotassiation in the initial two cycles. TEM and HRTEM images of Sb_2_MoO_6_ nanoplate b) being discharged to 0.80 V, c) being discharged to 0.01 V, and d) being charged to 2.70 V.

To profoundly validate the functions of Sb and Mo elements in the high‐capacity anode, the phase evolution portrayed by the contour plot of operando XRD results of Sb_2_MoO_6_/rGO composites during the initial two charge/discharge cycles is presented in **Figure** **4**a. Before the start of discharging process, distinctive XRD peaks from (202), (120), and (003) facets of Sb_2_MoO_6_ are detected. As the discharging process begins, those peaks turn out to be weaker and weaker, suggesting that the intercalation of K^+^ reduces the crystallinity of Sb_2_MoO_6_. At around 0.85 V, a peak around 28.5° emerges, which is assigned to (012) facet of Sb (PDF#85‐1322). It implies that Sb^3+^ in Sb_2_MoO_6_ is being reduced to Sb. As the potassiation process continues, a new peak close to 29.8° starts to show up around 0.57 V, which corresponds to (110) facet K_3_Sb (PDF#04‐0643). It is the product of the alloying reaction between Sb and K. In the following charging process, the peak from K_3_Sb gradually shifts to Sb peak near 0.62 V, pointing to the dealloying of K_3_Sb to yield Sb. However, the peaks of Sb_2_MoO_6_ are not recovered at all while the Sb peak sustains the position when the electrode is charged to 2.70 V, telling that the destruction of Sb_2_MoO_6_ lattice is irrecoverable, which explains the capacity difference between the theoretical capacity of Sb_2_MoO_6_ and the capacity achieved in this study. In the second cycle, the process of alloying and dealloying of Sb reiterates. It is worth mentioning that no XRD peaks associated with Mo based compounds are witnessed during the operando XRD measurements, signifying the production of amorphous Mo based compounds. The phase evolution of Sb_2_MoO_6_ during potassiation and depotassiation is further verified by the HRTEM images of Sb_2_MoO_6_ nanoplates at various discharging and charging status, as shown in Figure 4b–d. The electrodes for TEM images were being cycled for three times to achieve a more stable status and a small current density of 10 mA g^−1^ was implemented to approach the designated potential to reach the equilibrium state. In the TEM images of Sb_2_MoO_6_ nanoplates being discharged to 0.80 V (Figure 4b), a lattice spacing of 0.312 nm is identified, which is indexed to (012) facet of Sb, in agreement with operando XRD results. When the electrode is further discharged to 0.01 V (Figure 4c), a lattice distance of 0.303 nm is spotted, designated to (110) facet of K_3_Sb. Once the electrode is charged to 2.70 V (Figure 4d), the lattice spacing of (012) facet Sb returns. It is clear that the phase evolution obtained from TEM characterizations is in good agreement with operando XRD results. The corresponding TEM image, HAADF‐STEM image, and EDS mappings of Sb_2_MoO_6_ nanoplates at different discharging and charging states are shown in Figures S10–S12 (Supporting Information). They reveal the presence of Sb, Mo, and O, confirming that the nanostructures are discharged or charged product of Sb_2_MoO_6_ nanoplates. It also tells that Mo is still present in the discharged and charged electrodes, even if no Mo based compounds can be detected in the operando XRD characterization, ratifying the generation of amorphous Mo compound, which is designated as K–Mo–O considering the presence of K in the samples. The ratio of K:Mo:O acquired through the EDS quantitative analysis of TEM image is about 2.19:1:4.22, close to the possible product of K_2_O/MoO_3_. This amorphous matrix serves as a buffering matrix and alleviates the volume change of Sb during alloying and dealloying, thus enhancing the electrochemical stability of Sb_2_MoO_6_ electrode.

**Figure 5:**
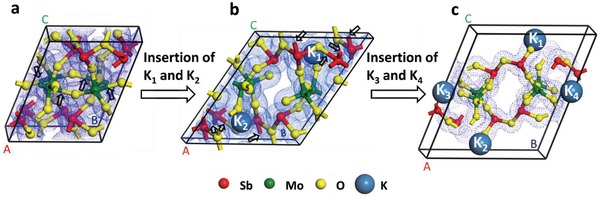
Lattice structure of a) pristine Sb_2_MoO_6_, b) with the insertion of a couple of potassium ions K_1_ and K_2_, and c) the insertion of two additional potassium ions K_3_ and K_4_.

To theoretically explore the K^+^‐insertion process into Sb_2_MoO_6_, DFT based calculations were employed using the Cambridge Serial Total Energy Package (CASTEP) plane wave code.^49^, ^50^, ^51^ The calculated equilibrium lattice parameters of Sb_2_MoO_6_ before and after the insertion of potassium ions K_1_ and K_2_ are shown in **Table** **1**. The initial lattice parameters of Sb_2_MoO_6_ after geometry optimization are consistent with the experimental results in the literature.^52^ Due to the large radius of K^+^ (1.38 Å), only a couple of symmetrical sites are suitable for the insertion of potassium ions. Meanwhile, the intercalation energy (*E*_i_) of potassium ions is computed according to the following equation

(1) $$E_{i}=E_{k+{\mathrm{Sb}}_{2}{\mathrm{MoO}}_{6}}-E_{k}-E_{{\mathrm{Sb}}_{2}{\mathrm{MoO}}_{6}}$$

where $E_{k+{\mathrm{Sb}}_{2}{\mathrm{MoO}}_{6}}$ and $E_{{\mathrm{Sb}}_{2}{\mathrm{MoO}}_{6}}$ are the total energies of potassium‐intercalated Sb_2_MoO_6_ and pristine Sb_2_MoO_6_, respectively, and *E*_k_ is the total energy of potassium. An *E*_i_ of −1.95 eV is obtained for the insertion of two potassium ions K_1_ and K_2_, which means the exothermic reaction and the attractive interaction occur, implying a favorable intercalation of these two potassium ions. After the geometry optimization of Sb_2_MoO_6_ with potassium ions K_1_ and K_2_ inside, the lattice constants of *b* and *c* noticeably expand, whereas the constant of *a* shrinks, as demonstrated in Table 1 and **Figure** **5**b. Since there are two potassium atoms in the end of crystal unit along BC direction, the lattice experiences a severe distortion in this direction, breaking four Mo—O bonds as shown by the arrows in Figure 5a and giving rise to a volume expansion around 23%. With the two potassium ions K_1_ and K_2_ lodged in the unit cell, another pair of sites appropriate for potassium ions to intercalate is created. With two additional potassium ions (K_3_ and K_4_) entering in the cell, the volume of the unit cell expands dramatically after the geometry optimization (Figure 5c), resulting in the fracture of Sb—O bonds (shown at the locations where the arrows point in Figure 5b). It is worth stating that the calculations are not able to converge after the addition of potassium ions K_3_ and K_4_ as the damage of Sb—O bonds happens. It is believed that more Sb—O and Mo—O bonds will be destructed with the insertion of more potassium ions, accompanied with the generation of Sb nanoparticles and amorphous Mo compounds. These DFT calculations are in line with results from operando XRD and TEM.

**Table 1:** Calculated equilibrium lattice parameters (*a*, *b*, and *c*) of Sb_2_MoO_6_ before and after the insertion of potassium ions K_1_ and K_2_, as well as the lattice parameters obtained from experimental results in the literature

Sb_2_MoO_6_	*a* [Å]	*b* [Å]	*c* [Å]	α [°]	β [°]	γ [°]	V [Å^3^]
Pristine	7.74	7.78	10.32	68.99	69.38	83.97	542.84
Experimental results of pristine^52^	7.48	7.50	10.12	70.43	70.91	83.35	505.84
With K_1_ and K_2_	7.23	9.81	11.44	58.63	77.76	91.57	667.69

**Figure 6:**
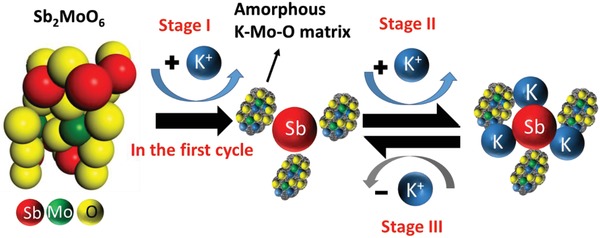
The proposed route for the potassiation/depotassiation process in Sb_2_MoO_6_.

From the above analysis, the full potassiation/depotassiation course of Sb_2_MoO_6_ can be summarized in **Figure** **6**, which could be divided into three stages as follows. In the stage I, the lattice of Sb_2_MoO_6_ is destructed upon the intercalation of K^+^ during the discharging process in the first cycle, with the Mo bonds broken first and the Sb—O bonds shattered later. Sb^3+^ in Sb_2_MoO_6_ is reduced to Sb while Mo element forms an amorphous compound of K—Mo—O, possibly K_2_O/MoO_3_. In the continuous discharging process (stage II), alloying reaction between Sb and K^+^ occurs, producing K_3_Sb. In the final period (stage III), the dealloying process of K_3_Sb takes place and Sb is regenerated in the charging process. The stage I is irreversible, but stage II and stage III are reversible. Therefore, it is concluded that Sb element is primarily responsible for the high capacity for Sb_2_MoO_6_ anode, while Mo element improves the conductivity and forms an amorphous matrix that improves the cyclability of the electrode

(I) $${\mathrm{Sb}}_{2}{\mathrm{MoO}}_{6}+K^{+}+e\to \mathrm{Sb}+\mathrm{K‐Mo‐O}\left(\mathrm{possibly}\;K_{2}{\mathrm{O/MoO}}_{3}\right)$$

(II) $$\mathrm{Sb}+K^{+}+e\to K_{3}\mathrm{Sb}$$

(III) $$K_{3}\mathrm{Sb}-K^{+}-e\to \mathrm{Sb}$$

## 3 Conclusion

In summary, Sb_2_MoO_6_/rGO composites were successfully synthesized as a high‐performance anode material for KIBs through a facile hydrothermal method. This material achieved a discharge capacity as high as 402 mAh g^−1^ at a current density of 100 mA g^−1^ and 381 mAh g^−1^ at 200 mA g^−1^, and a discharge capacity of 247 mAh g^−1^ was preserved after 100 cycles at a large current density of 500 mA g^−1^ with a high Columbic efficiency. In combination of the electrochemical techniques, operando XRD, TEM, and DTF calculation, it can be deduced that the majority of the high‐capacity anode is from Sb, which stems from the irreversible destruction of Sb_2_MoO_6_ lattice, with the Mo bonds broken first and the Sb—O bonds destructed later, followed by the reduction of Sb^3+^. Sb nanoparticles are able to alloy and dealloy with K^+^ to offer a high capacity, while Mo element not only improves the conductivity but also forms an amorphous matrix that relieves the volume change of Sb during charge/discharge, effectively promoting the rate performance and cycling performance. This study sheds light on the nature of potassium‐storage mechanism of Sb_2_MoO_6_ and provides sight into the fundamental study on metal‐oxide based electrode materials for KIBs, which is vital in the development of KIBs.

## 4 Experimental Section

*Material Synthesis*: Sb_2_MoO_6_/rGO composites were synthesized using a hydrothermal method. First, GO nanosheets, which were fabricated through a modified Hummer's method,^53^ were dispersed in deionized water (20 mL) via ultrasonication in a concentration of 0.5 mg mL^−1^. Then Na_2_MoO_4_ · 2H_2_O (1 mmol) (≥99.0%, Sinopharm Chemical Reagent Co., Ltd) was completely dissolved in the above solution through magnetic stirring to serve as the Mo source. Thereafter, a solution with SbCl_3_ (2 mmol) (≥99.0%, Sinopharm Chemical Reagent Co., Ltd) dissolved in ethanol (5 mL) was added into Na_2_MoO_4_/GO solution dropwise under a vigorous stirring. After being magnetically stirred for 30 min, the mixtures were transferred to a Teflon autoclave and then let it stay at 180 °C for 24 h. The obtained greenish products were rinsed with deionized water and ethanol for several times. The final products were separated by centrifugation and dried with freeze drying. The identical fabrication process was applied for preparing Mo‐based and Sb‐based counterparts, except for that SbCl_3_ was not available for the Mo‐based counterpart while Na_2_MoO_4_ was excluded for Sb‐based counterpart.

*Material Characterizations*: AFM was performed on a Park XE7 AFM. XRD results were acquired by Bruker D8 ADVANCE (Cu Kα). With the investigated material coated on an Al foil as the working electrode, operando XRD experiments were run at a current density of 50 mA g^−1^ using a potassium foil as the reference and counter electrode and an electrolyte of 3 m KFSI in DME. A field emission scanning electron microscope (Hitachi S‐4800) was adopted to characterize the morphology. The detailed lattice information and elemental distribution were obtained through a Titan G2 60–300 TEM. Raman spectra were recorded using a Renishaw Raman instrument (InVia Raman Microscope) with a green laser at 532 nm.

*Electrochemical Measurements*: Electrodes with Sb_2_MoO_6_/rGO composites, Sb‐based counterpart (Sb_2_O_3_/Sb_8_O_11_Cl_2_/rGO composites), and Mo‐based counterpart (MoO_2_/rGO composites) were fabricated via mixing the active materials, acetylene black, and carboxymethyl cellulose in a solution of C_2_H_5_OH and H_2_O with a mass ratio of 8:1:1. Then the mixtures were spread onto a piece of copper foil with an average loading around 1.0 mg cm^−2^. 2032 coin cells were utilized which were assembled in a glovebox filled with Ar. The explored material served as the working electrode, a piece of potassium metal functioned as the reference electrode and counter electrode, and 3 m KFSI dissolved in DME was the electrolyte. Approximately 80 µL electrolyte was applied for every coin cell. To obtain a more densely packed SEI film, the cells were charged and discharged at 100 mA g^−1^ for three times before the cycling tests at 500 mA g^−1^ for all three materials. The battery performance was evaluated by Neware BTS‐53 in a voltage range of 0.01–2.70 V. CV and EIS were executed using a CHI electrochemical workstation. The cycled cells with Sb_2_MoO_6_ nanoplate electrodes were cautiously disassembled and rinsed by DME in the glovebox to investigate Sb_2_MoO_6_ nanoplates at various charge and discharge status.

*Computational Section*: Ultrasoft pseudopotentials were adopted to describe the interaction between ionic core and valence electrons. Valence states in this study were considered in regard to O2s^2^2p^4^, Sb5s^2^5p^3^, Mo4d^5^5s^1^, and K3p^6^4s^1^. The Perdew–Burke–Ernzerhof generalized gradient approximation method parameterized by Perdew was selected to analyze the exchange and correlation terms.^54^, ^55^ Brillouin‐zone integrations were performed using Monkhorst and Pack *k*‐point meshes.^56^ During the calculation of Sb_2_MoO_6_, a cutoff energy of 380 eV and a k‐point number of 3 × 3 × 3 were applied to ensure the convergence of the total energy. All calculations were converged when the maximum force on the atom was below 0.01 eV Å^−1^, the maximum stress was below 0.02 GPa, and the maximum displacement between cycles was below 0.0005 Å.

## Conflict of Interest

The authors declare no conflict of interest.

## Supporting information

Supplementary
