# Supplementary material for: Nature of Bimetallic Oxide Sb2MoO6/rGO Anode for High‐Performance Potassium‐Ion Batteries
Source: Adv Sci (Weinh). 2019 Jun 18;6(17):1900904. doi: 10.1002/advs.201900904 (PMC6724349; doi:10.1002/advs.201900904)
Supplement: Supplementary file 1 — Supplementary [file ADVS-6-1900904-s001.pdf]

Copyright WILEY-VCH Verlag GmbH & Co. KGaA, 69469 Weinheim, Germany, 2019.

ADVANCED SCIENCE
Open Access

Supporting Information

for *Adv. Sci.,* DOI: 10.1002/advs.201900904

Nature of Bimetallic Oxide Sb_2MoO_6/rGO Anode for High-Performance Potassium-Ion Batteries

*Jue Wang, Bin Wang, Zhaomeng Liu, Ling Fan, Qingfeng Zhang, Hongbo Ding, Longlu Wang, Hongguan Yang, Xinzhi Yu, and Bingan Lu**

WILEY-VCH

Supporting Information

Nature of Bimetallic Oxide Sb_2MoO_6/rGO Anode for High-Performance Potassium Ion Batteries

*Jue Wang, Bin Wang, Zhaomeng Liu, Ling Fan, Qingfeng Zhang, Hongbo Ding, Longlu Wang, Hongguan Yang, Xinzhi Yu, and Bingan Lu**

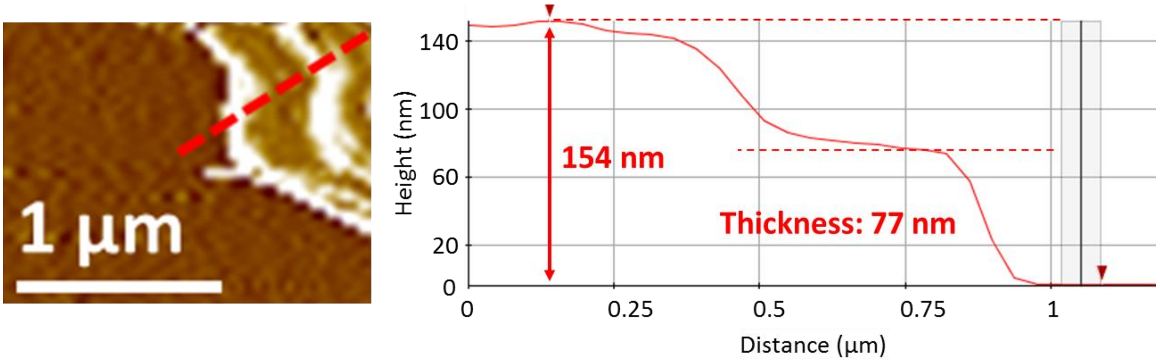

Figure S1. AFM image and the corresponding height profile of Sb_2MoO_6 nanoplates.

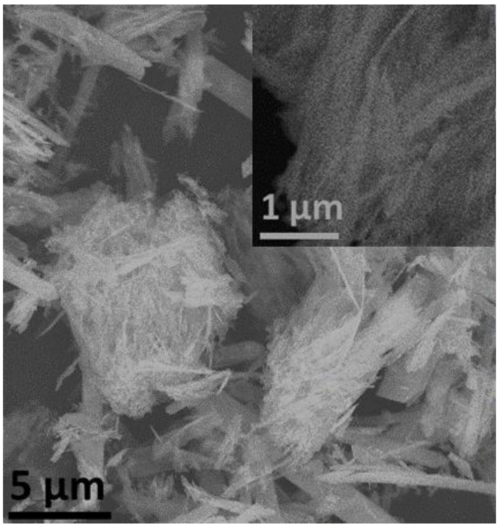

Figure S2. SEM images of Sb_2MoO_6 synthesized without the addition of GO during the synthesis.

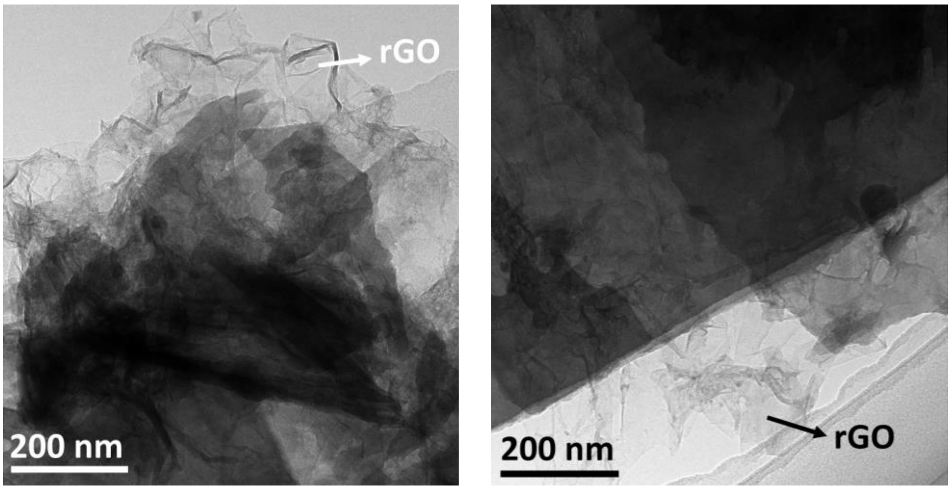

Figure S3. TEM images of Sb_2MoO_6 nanoplates with rGO nanosheets.

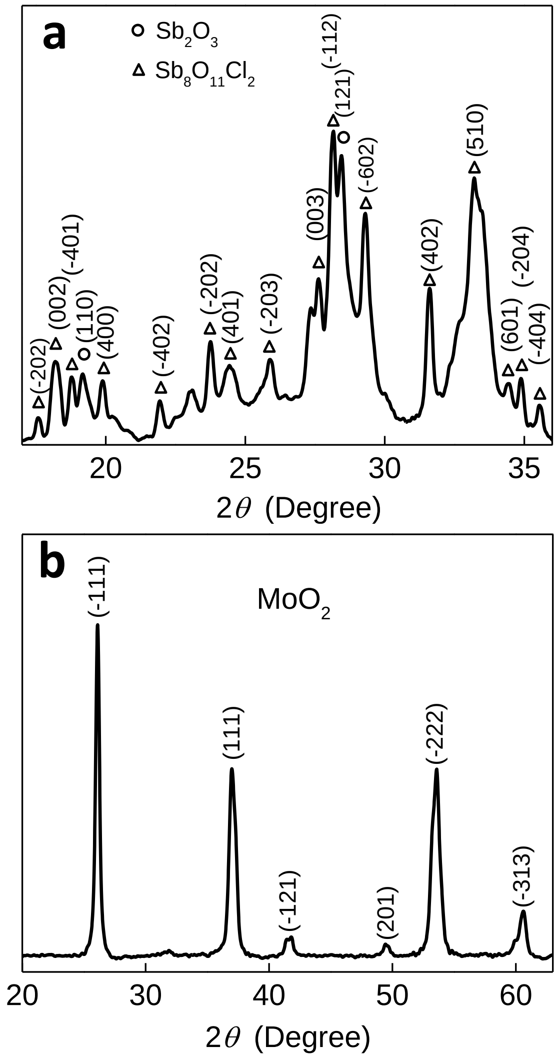

Figure S4. XRD patterns of a) Sb-based counterpart (Sb_2O_3/$Sb_8O_{11}Cl_2$/rGO) and b) Mo-based counterpart (MoO_2/rGO).

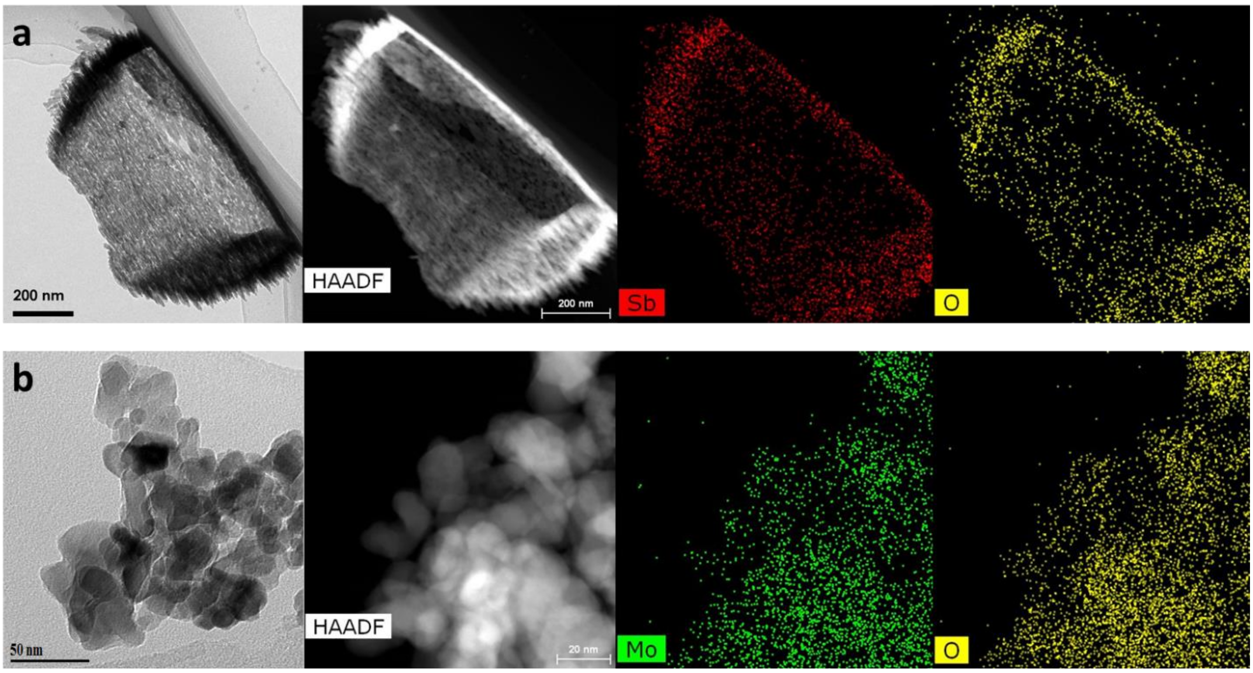

Figure S5. TEM images, HAADF-STEM images, and EDS mappings of a) $Sb_2O_3/Sb_8O_{11}Cl_2$ nanoplate/rGO and b) MoO_2 nanoplate/rGO.

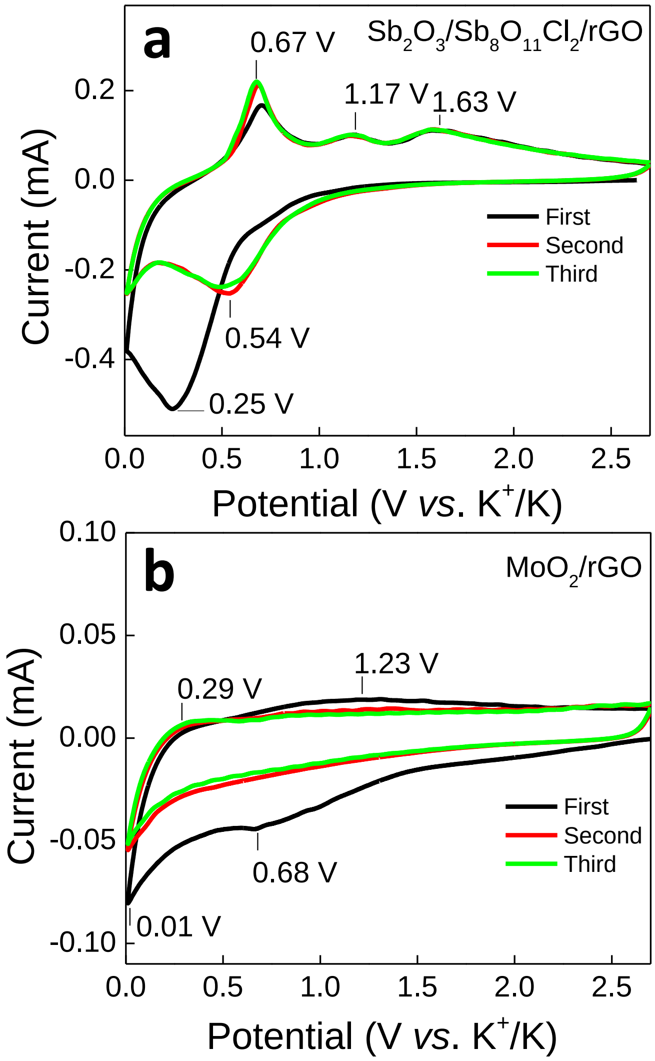

Figure S6. CV curves of a) $Sb_2O_3/Sb_8O_{11}Cl_2$/rGO and b) MoO_2/rGO.

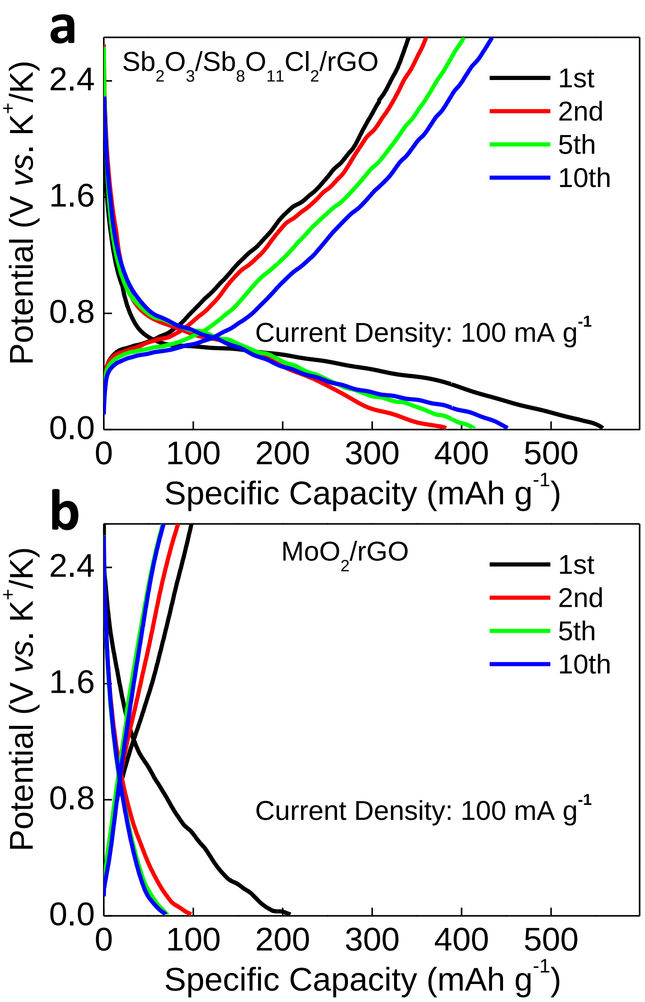

Figure S7. Charge/discharge profiles of a) $Sb_2O_3/Sb_8O_{11}Cl_2$/rGO and b) MoO_2/rGO.

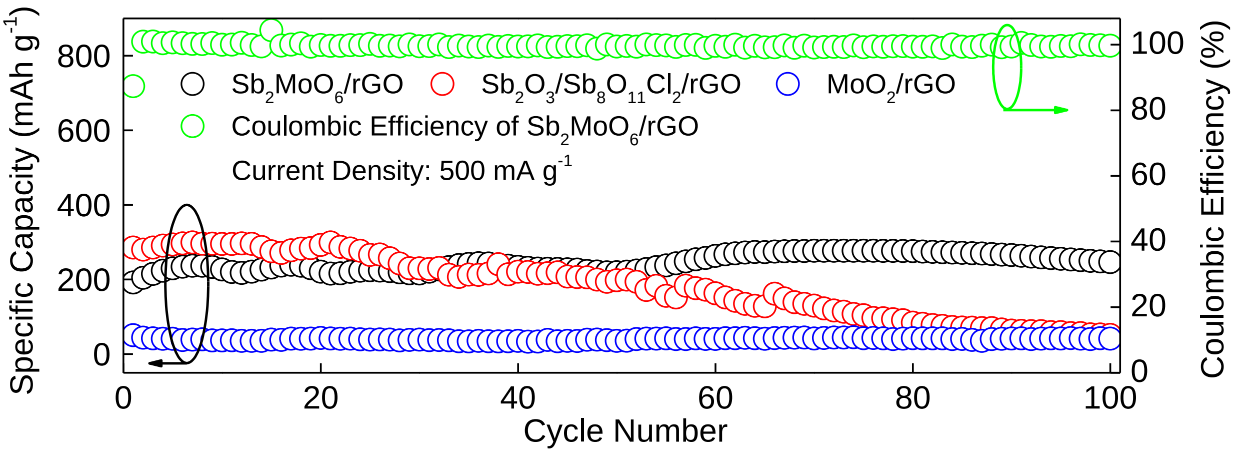

Figure S8. Cycling performance of Sb_2MoO_6/rGO, $Sb_2O_3/Sb_8O_{11}Cl_2$/rGO, and MoO_2/rGO at 500 mA g^{-1}.

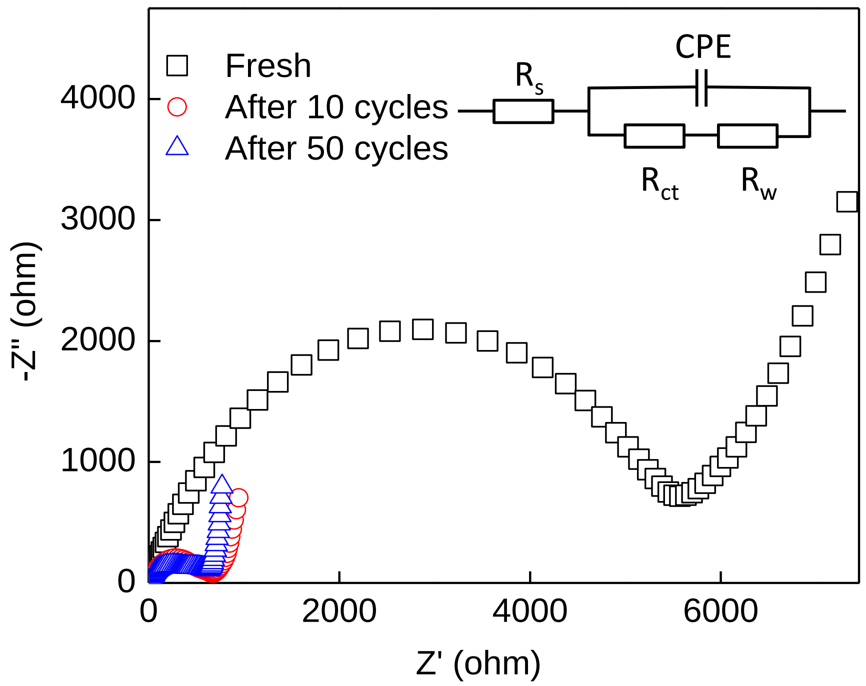

Figure S9. Nyquist plots of the Sb_2MoO_6/rGO electrode, which is fresh, after 10 cycles at 500 mA g^{-1}, and after 50 cycles at 500 mA g^{-1}, with the equivalent-circuit model inside.

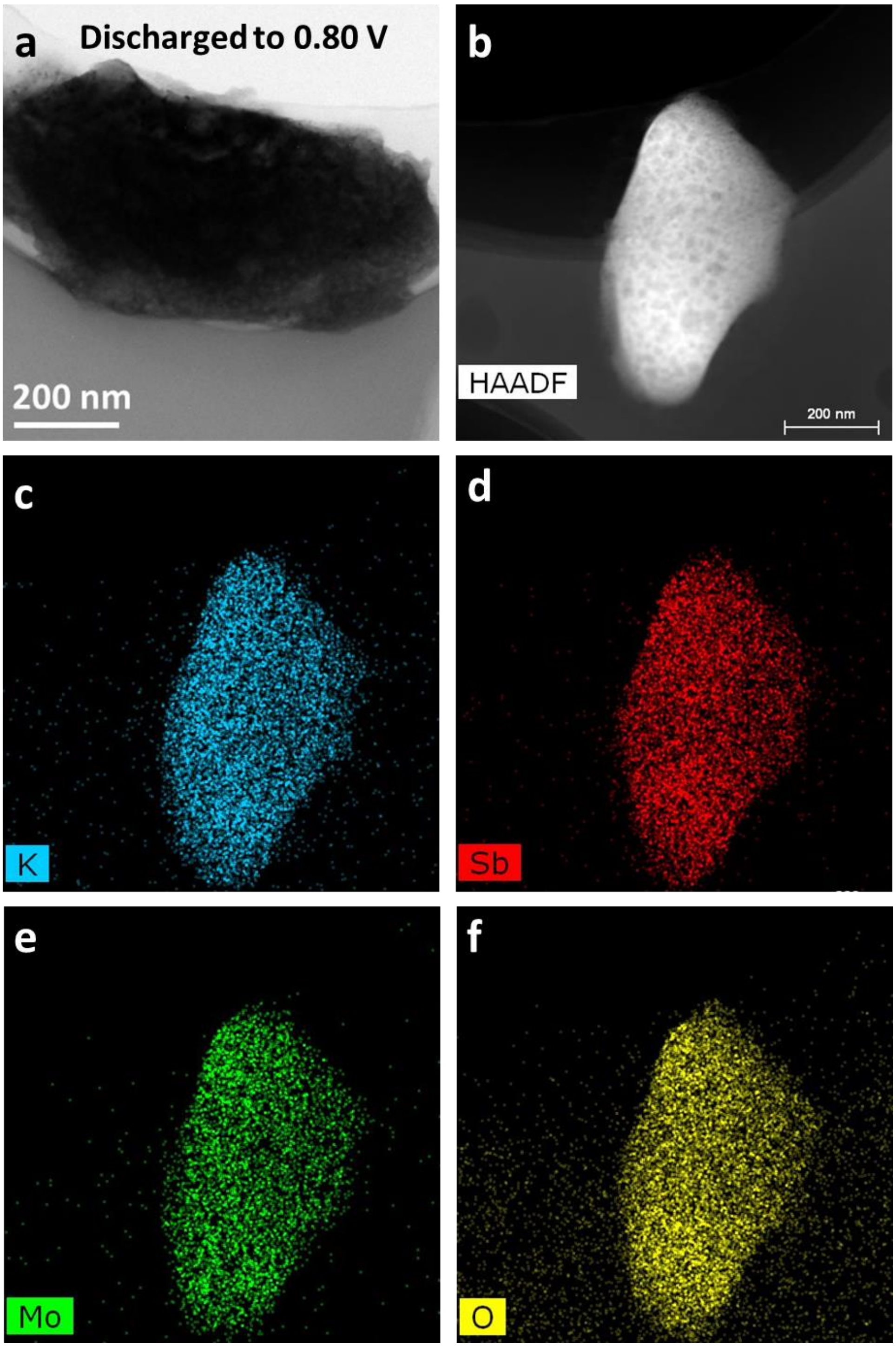

Figure S10. a) TEM image, b) HAADF-STEM image, and c–f) EDS mappings of Sb_2MoO_6 nanoplate being discharged to 0.80 V.

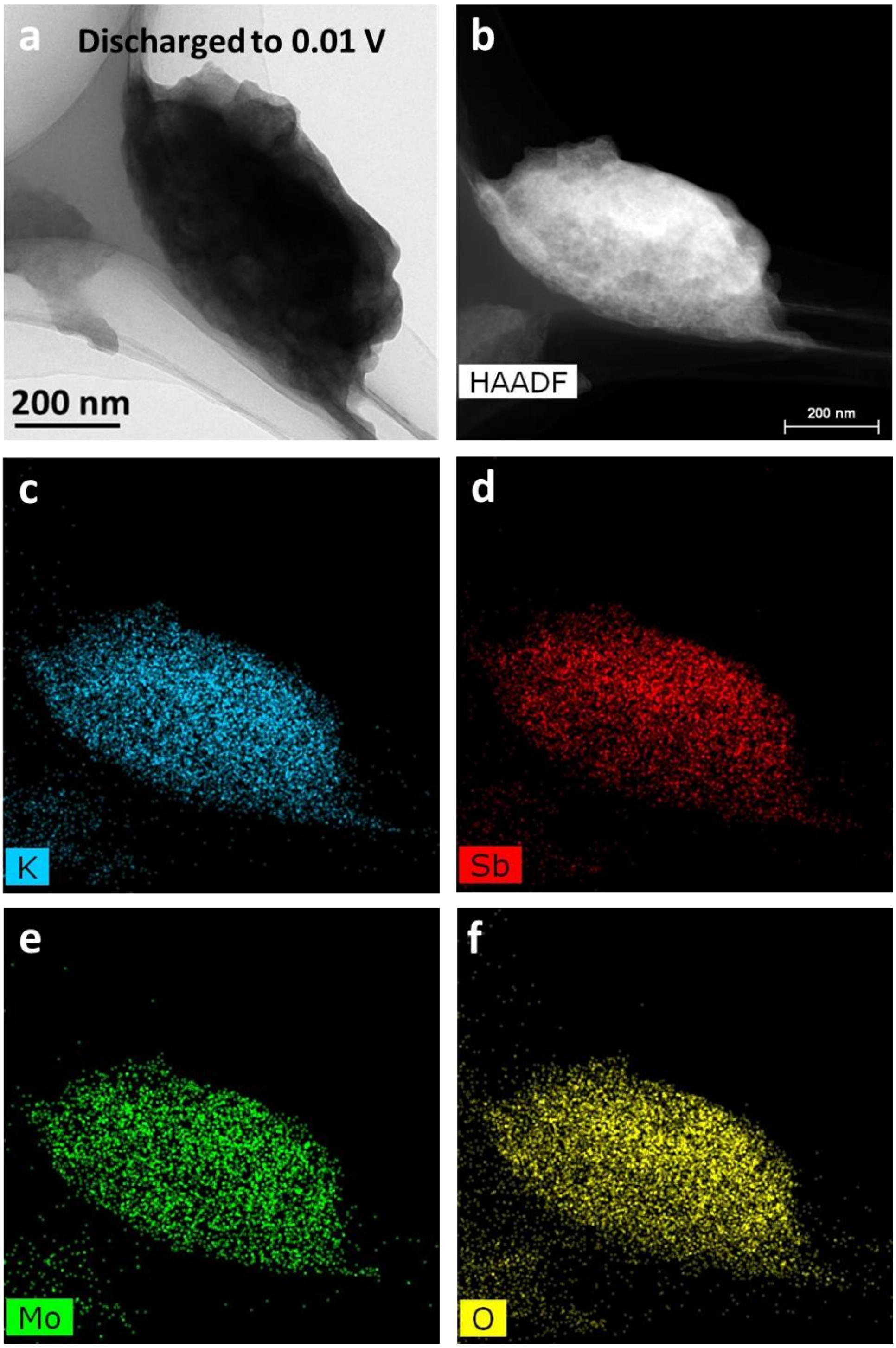

Figure S11. . a) TEM image, b) HAADF-STEM image, and c–f) EDS mappings of Sb_2MoO_6 nanoplate being discharged to 0.01 V.

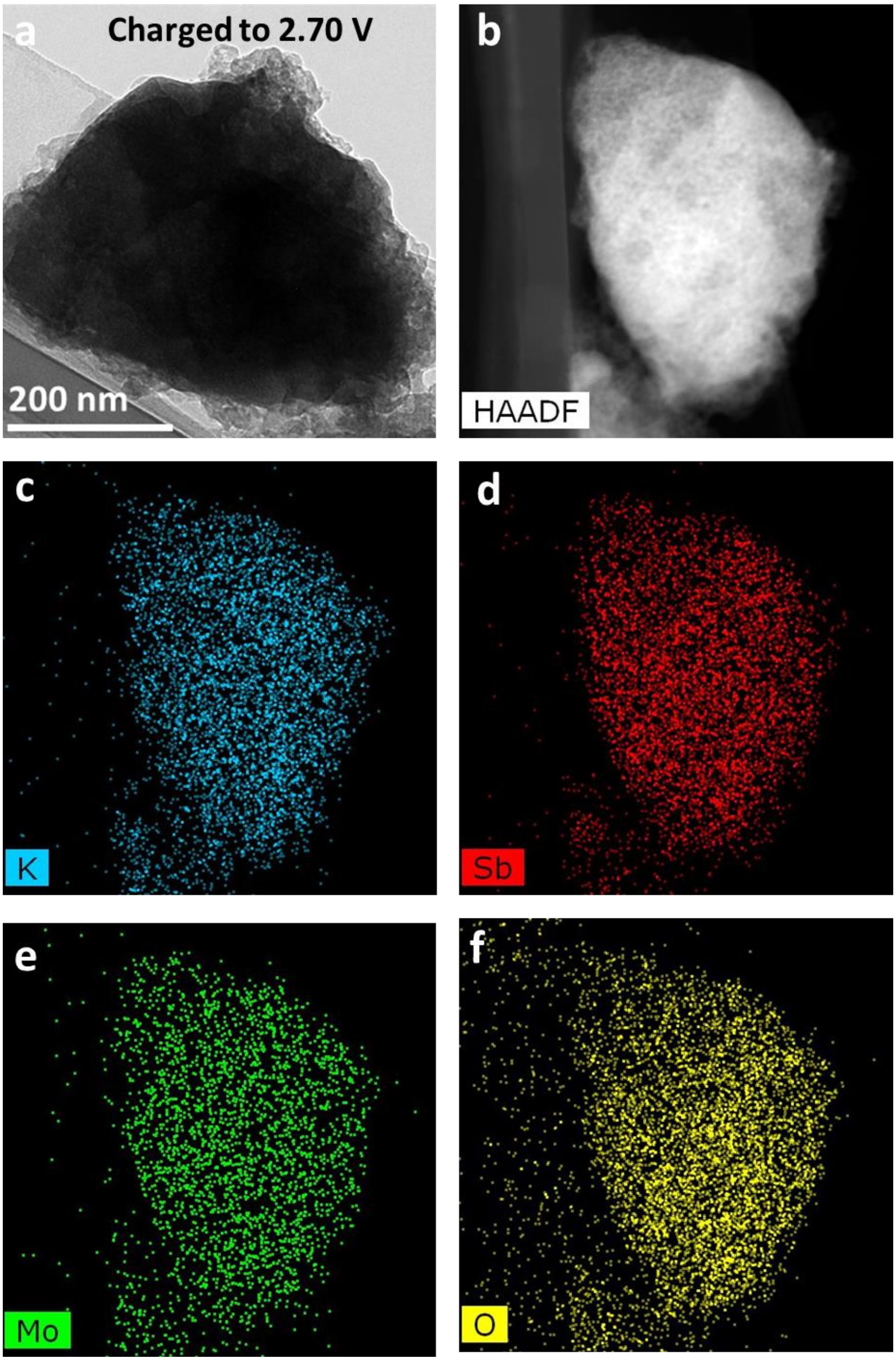

Figure S12. a) TEM image, b) HAADF-STEM image, and c–f) EDS mappings of Sb_2MoO_6 nanoplate being charged to 2.70 V.
